# A Coordinated Project-Management Approach to Multisite Implementation of Motor-Rehabilitation Programs for Children With Autism Spectrum Disorder in United States Healthcare Systems: A Narrative Review

**DOI:** 10.7759/cureus.100513

**Published:** 2025-12-31

**Authors:** Amienye B Omo Enabulele, Iyiola Oyebamiji, Opeyemi Ikubanni, Micah Nnabuko Okwah, Wuraola Susan Babalola, Oyebisi M Azeez, Aliyu O Olaniyi

**Affiliations:** 1 College of Business, Missouri State University, Springfield, USA; 2 Sociology, University of Ilorin, Ilorin, NGA; 3 Sociology/Criminology, University of Ilorin, Ilorin, NGA; 4 Public Health, Yale School of Public Health, New Haven, USA; 5 Carey Business School, Johns Hopkins University, Baltimore, USA; 6 Veterinary Physiology and Biochemistry, University of Ilorin, Ilorin, NGA; 7 Geriatrics, Stepping Hill Hospital, Stockport, GBR

**Keywords:** autism spectrum disorder, healthcare systems, health equity, implementation science, motor rehabilitation, multisite coordination, pediatric neurorehabilitation, project management, telehealth, translational research

## Abstract

Motor impairments are common in children with autism spectrum disorder (ASD) and contribute to reduced functional independence, participation, and quality of life. Although motor-rehabilitation interventions can improve motor and adaptive outcomes, services are often fragmented, inconsistently delivered, and difficult to scale beyond single clinical settings. Multisite implementation of ASD motor-rehabilitation programs remains limited, underscoring the need for coordinated approaches that address both clinical and organizational challenges.

This integrative narrative review synthesizes representative literature on motor-rehabilitation interventions for children with ASD alongside implementation-science and project-management frameworks relevant to multisite healthcare delivery in the United States. A broad search of peer-reviewed literature and policy sources published between 2000 and 2025 was conducted. Evidence was synthesized qualitatively with attention to implementation-relevant factors, governance structures, and scalability considerations; no quantitative synthesis or statistical analysis was performed.

The reviewed literature indicates that motor-rehabilitation interventions can improve motor proficiency, adaptive behavior, and participation in children with ASD. However, the evidence base is dominated by small, single-site studies with substantial heterogeneity in intervention design and outcome reporting. Key barriers to multisite implementation include variability in workforce training, infrastructure, reimbursement models, data systems, and family engagement across settings. Implementation-science frameworks (e.g., the Consolidated Framework for Implementation Research (CFIR), Reach-Effectiveness-Adoption-Implementation-Maintenance (RE-AIM), and Normalization Process Theory) and project-management methodologies offer complementary strategies to address these barriers but are rarely integrated within ASD motor-rehabilitation research.

Drawing on these domains, this review proposes a Coordinated Project-Management Framework to guide multisite implementation through iterative phases of initiation, planning, delivery, monitoring and evaluation, and sustainability. Scaling effective motor-rehabilitation services for children with ASD will require coordinated, multisite implementation rather than isolated clinic-based efforts. Future research should prioritize piloting and evaluating this framework in real-world healthcare networks using hybrid effectiveness-implementation designs to improve access, consistency, and equity in ASD motor rehabilitation.

## Introduction and background

Motor impairments are common in children with autism spectrum disorder (ASD) and have meaningful consequences for functional independence, participation in physical and social activities, and overall quality of life [1,2]. Despite this, ASD services have historically prioritized communication and behavioral challenges, often leaving motor difficulties under-recognized and inconsistently addressed. Motor rehabilitation, encompassing physical therapy (PT), occupational therapy (OT), and related sensorimotor interventions, therefore represents an essential but under-integrated component of comprehensive ASD care [3,4].

Evidence indicates that motor-rehabilitation interventions can improve motor proficiency, adaptive behavior, and social engagement in children with ASD [3,4]. However, most studies are small, single-site trials conducted under controlled conditions, with substantial variability in intervention content, outcome measures, and fidelity reporting. These limitations reduce generalizability and complicate translation into routine practice. When interventions are extended beyond individual clinics, additional barriers emerge, including differences in infrastructure, workforce training, reimbursement models, and family engagement practices [5]. As a result, effective ASD motor-rehabilitation programs often fail to spread beyond isolated settings.

The urgency of scalable solutions is increasing. ASD prevalence in the United States has risen substantially over the past two decades, with current estimates indicating that approximately one in 36 children is affected [6,7]. National initiatives such as the Autism Collaboration, Accountability, Research, Education, and Support Act (Autism CARES Act) and the NIH Autism Centers of Excellence (ACE) network have expanded research and service capacity [8]. However, these investments have not consistently translated into coordinated, multisite delivery of motor-rehabilitation services across healthcare, educational, and community-based systems.

Addressing this gap requires more than additional evidence of clinical effectiveness. Multisite implementation of ASD motor-rehabilitation programs demands deliberate coordination of people, processes, data, and resources across heterogeneous settings. Implementation science clarifies the factors that influence adoption, fidelity, and sustainability, while project-management approaches provide practical tools for organizing the planning, execution, monitoring, and sustainability of multisite initiatives [9]. To date, however, these domains have rarely been integrated in a manner tailored to ASD motor rehabilitation.

Accordingly, this narrative review focuses specifically on motor rehabilitation in children with ASD, with an emphasis on the challenges and requirements of multisite implementation. The objectives are to (1) synthesize representative evidence on ASD motor-rehabilitation interventions with attention to implementation-relevant factors; (2) examine implementation-science and project-management models applicable to multisite healthcare initiatives; and (3) present a practical, coordinated project-management framework tailored to multisite implementation of motor-rehabilitation programs for children with ASD.

By integrating clinical evidence with applied implementation and project-management strategies, this review aims to move beyond descriptive summaries and provide an ASD-specific roadmap for translating effective motor-rehabilitation interventions into scalable, real-world practice.

## Review

Methodology

Study Design

This study is an integrative narrative review examining multisite implementation of motor-rehabilitation services for children with ASD within U.S. healthcare systems. An integrative narrative approach was selected to enable conceptual synthesis across clinical, implementation-science, and project-management literature rather than to provide an exhaustive or strictly reproducible mapping of all eligible studies.

Accordingly, this review prioritizes transparency of scope, conceptual coherence, and translational relevance over formal systematic-review procedures such as the Preferred Reporting Items for Systematic Reviews and Meta-Analyses (PRISMA) flow accounting or quantitative risk-of-bias appraisal. In keeping with the integrative narrative design, no statistical summary methods were applied. Specifically, no meta-analysis, meta-regression, or pooled quantitative synthesis was conducted, and no inferential statistics such as P-values or confidence intervals were generated.

Search Strategy

A broad and systematic search strategy was used to support narrative synthesis. Searches were conducted in PubMed/MEDLINE, Scopus, PsycINFO, CINAHL, and Web of Science for publications between January 2000 and September 2025. Search terms combined ASD, motor rehabilitation (e.g., PT, OT, sensorimotor intervention), multisite or scalable healthcare delivery, implementation science, and project management.

Reference lists of relevant systematic reviews and key empirical studies were screened to identify additional sources. Grey literature and policy documents from U.S. federal agencies (e.g., the Centers for Disease Control and Prevention (CDC), the National Institutes of Health (NIH), and the Health Resources and Services Administration (HRSA)) were reviewed to contextualize findings within national ASD research and service initiatives. These documents were used for contextual and policy framing rather than as sources of clinical efficacy evidence.

Search terms were applied in flexible combinations rather than as fixed, fully reproducible strings, reflecting the integrative narrative design of the review. Core concepts included ASD, motor rehabilitation, PT, OT, and motor intervention, combined with terms related to multisite implementation, scalability, implementation science, project management, and digital health. Searches were limited to English-language publications and pediatric populations where applicable.

Eligibility Criteria

Sources were included if they met at least one of the following criteria: (1) ASD-specific motor-rehabilitation studies involving children or adolescents, including PT, OT, or sensorimotor interventions; (2) pediatric or neurodevelopmental rehabilitation studies providing implementation-relevant insights applicable to multisite delivery; or (3) implementation-science, project-management, or digital-health literature offering conceptual or operational guidance for coordinating complex multisite healthcare initiatives.

Studies focused exclusively on pharmacological treatments, non-motor interventions, or adult-only populations without relevance to pediatric rehabilitation were excluded.

ASD-specific motor-rehabilitation studies form the core clinical evidence base of this review. Literature from other domains is included strictly as contextual or conceptual support and is not presented as evidence of clinical efficacy for children with ASD.

Data Extraction and Synthesis

Data extraction was conducted by a single reviewer using a structured framework capturing study population, intervention type, setting, and implementation-relevant characteristics. Interpretive decisions and thematic categorizations were reviewed and discussed among co-authors to ensure conceptual coherence.

Given the integrative narrative design, no formal risk-of-bias assessment of individual studies was conducted. Instead, study quality was considered narratively, with attention to design characteristics such as randomized versus non-randomized designs, sample size, multisite versus single-site implementation, and clarity of intervention and outcome reporting.

Quantitative aggregation was not undertaken because of substantial heterogeneity across the reviewed studies, including differences in participant characteristics, intervention content and intensity, outcome measures, follow-up duration, and study design. This heterogeneity precluded meaningful pooling of effect sizes and supports a qualitative, narrative synthesis as the most appropriate analytic approach.

Accordingly, findings from individual studies are summarized descriptively and comparatively, with attention to the direction and general magnitude of reported motor outcomes, rather than through formal statistical synthesis. Where systematic reviews or meta-analyses are cited, their conclusions are referenced narratively to contextualize the evidence base. The Scale for the Assessment of Narrative Review Articles (SANRA) was used solely to guide reporting quality and transparency of the narrative review itself, and not to appraise or grade the quality of individual primary studies.

While the methods described support transparency and conceptual traceability, the narrative design inherently limits strict reproducibility, and the findings should be interpreted as a translational synthesis rather than a replicable systematic review.

Review

Motor Function and Rehabilitation in ASD

Motor impairments are increasingly recognized as a common and clinically meaningful feature of ASD, despite not being included in the core diagnostic criteria. A substantial proportion of children with ASD experience delays or deficits in gross motor coordination, fine-motor control, balance, gait, and motor planning, which can limit independence and participation in everyday activities such as self-care, play, and school-related tasks [10]. These motor difficulties meaningfully influence functional outcomes and therefore represent important targets for intervention rather than ancillary features of ASD.

Neurological and developmental context: Neuroimaging and neurophysiological studies implicate differences in cerebellar, basal ganglia, and cortical motor networks in children with ASD, alongside atypical connectivity and altered sensorimotor integration [11]. Many children also meet criteria for co-occurring motor conditions such as developmental coordination disorder (DCD) and frequently present with attentional or executive-function difficulties associated with attention-deficit/hyperactivity disorder (ADHD), further complicating motor learning and performance. Sensory processing differences, particularly involving proprioceptive and vestibular systems, may interfere with the acquisition and generalization of motor skills [12].

From an implementation perspective, these findings highlight that effective motor-rehabilitation programs for ASD must address not only isolated motor skills, but also sensory integration, attention, and learning conditions in a consistent manner across sites. Importantly, neural and developmental profiles vary widely across the autism spectrum, influenced by factors such as symptom severity, co-occurring conditions, and developmental stage, reinforcing the need for structured yet adaptable rehabilitation approaches.

Functional and psychosocial implications: Motor impairments in ASD have direct implications for academic participation, social inclusion, and long-term health. Fine-motor difficulties can interfere with handwriting, tool use, and classroom engagement, while gross-motor limitations may restrict participation in sports and community activities, contributing to social isolation and sedentary behavior. Improvements in motor competence are therefore associated with downstream benefits in participation, self-efficacy, and overall quality of life [13].

At the programmatic level, these functional outcomes can inform the selection of implementation-relevant performance indicators for multisite motor-rehabilitation initiatives. Improvements in motor competence may be captured through standardized motor assessments, gains in functional autonomy through measures of daily living skills or caregiver reports, and participation through school or community engagement metrics. Consistency in outcome measurement, intervention fidelity, and reach across sites further supports coordinated evaluation and continuous quality improvement in multisite settings.

Rationale for structured motor-rehabilitation programs: Given the prevalence and functional impact of motor impairments in ASD, structured motor-rehabilitation interventions delivered through PT, OT, or integrated sensorimotor approaches represent a key opportunity to improve outcomes [14]. However, current service delivery is often fragmented, with substantial variability in intervention content, intensity, and access across clinical and community settings. These inconsistencies limit scalability and equity of care.

For the purposes of this review, core components of motor rehabilitation in ASD are defined as: (1) systematic assessment of gross and fine motor function using standardized tools; (2) individualized, goal-oriented intervention targeting coordination, balance, strength, and motor planning; (3) integration of sensory and attentional supports to facilitate motor learning; (4) structured opportunities for practice and generalization across home, school, and community contexts; and (5) ongoing monitoring of progress and fidelity to guide adaptation over time. Together, these components provide a shared clinical foundation upon which multisite implementation and coordinated project-management strategies can be applied.

Representative Evidence Informing Motor-Rehabilitation and Multisite Implementation in ASD

Recent systematic reviews and meta-analyses report overall positive effects of motor and physical-activity interventions on motor competence in children with ASD, while also highlighting substantial heterogeneity in intervention design, outcome measures, and reporting quality [5,15]. A broad range of motor-rehabilitation approaches has been studied, including conventional therapy models, play- and socially oriented interventions, technology-assisted modalities, and emerging tele-rehabilitation and hybrid models [5]. Although findings are generally promising, most studies are limited by small sample sizes, short follow-up periods, and limited attention to implementation processes.

From a multisite perspective, evidence of clinical benefit alone is insufficient. Effective scaling requires a clear specification of what intervention components are delivered, by whom, under what conditions, and with what level of fidelity. Accordingly, this section synthesizes representative ASD-specific evidence while drawing selectively on related pediatric rehabilitation and digital-health literature to inform implementation considerations, rather than to extend ASD-specific efficacy claims. Across intervention modalities, consistent elements relevant to multisite delivery include structured, goal-directed motor practice; opportunities for repetition and feedback; caregiver or contextual support to promote generalization; and the use of standardized outcome measures and fidelity monitoring.

Conventional PT and OT: Conventional PT and OT remain the most commonly delivered motor-rehabilitation interventions for children with ASD. These approaches typically emphasize repetitive practice of targeted motor skills, such as balance, strength, bilateral coordination, and fine-motor control, and often incorporate sensory-based strategies to support modulation and body awareness [3]. Controlled studies report improvements in motor proficiency and postural control, although effect sizes and outcome measures vary.

At the implementation level, PT and OT programs differ widely in session frequency, duration, therapist training, and family involvement across clinical sites. For coordinated multisite implementation, a clearer definition of core intervention components, recommended intensity ranges, and standardized outcome measures is needed, along with explicit strategies for caregiver engagement and fidelity monitoring [16].

Play-based and social-motor interventions: Play-based and social-motor interventions embed motor goals within structured or semi-structured play activities, including motor games, peer-based programs, and reciprocal imitation tasks [17]. These approaches align with developmental models emphasizing the interdependence of movement, social interaction, and communication. Studies suggest improvements in motor coordination, imitation, and participation, although methodologies and outcome measures are heterogeneous.

Multisite implementation of play-based interventions often spans healthcare and educational contexts, such as schools and early intervention programs. This requires aligned training for therapists and educators, shared goal-setting processes, and mechanisms to monitor fidelity and outcomes consistently across sites [18].

Technology-assisted and tele-rehabilitation models: Technology-assisted motor-rehabilitation approaches, including virtual reality systems, motion-based gaming, and robotic-assisted training, offer opportunities for high-repetition practice with immediate feedback and increased engagement [19]. Early studies support feasibility and potential motor benefits, but most evaluations are small and conducted in specialized settings.

Tele-rehabilitation and hybrid delivery models, which expanded during the COVID-19 pandemic, enable remote coaching and home-based motor practice, increasing reach for families in rural or underserved areas [20]. These models introduce additional implementation challenges related to digital access, caregiver training, technical support, and cross-site data integration. Evidence from broader pediatric rehabilitation and digital-health contexts informs these implementation considerations, but direct extrapolation to ASD populations should be made cautiously due to differences in communication, sensory profiles, and caregiver involvement.

Evidence gaps relevant to multisite implementation: To date, most motor-rehabilitation interventions for children with ASD have been evaluated in single-site or small controlled studies. True multisite evaluations are rare and typically focus on feasibility or early implementation outcomes rather than large-scale effectiveness [5]. Several recurring gaps limit scalability: inconsistent intervention descriptions that hinder replication; non-standardized outcome measures that impede comparison across sites; and minimal reporting of implementation outcomes such as adoption, fidelity, reach, and sustainability [21].

These limitations highlight the need for structured approaches that integrate clinical content with implementation planning and coordination. Table 1 summarizes representative studies and highlights implementation-relevant insights, including challenges related to fidelity, measurement, and scalability. Taken together, the evidence suggests that improving outcomes in ASD motor rehabilitation will require not only stronger clinical trials but also systematic implementation and project-management strategies to support adoption, delivery, and sustainability across multiple sites.

**Table 1:** Representative autism spectrum disorder (ASD)-specific studies and illustrative cross-domain examples informing multisite motor-rehabilitation implementation ASD-specific studies are presented as representative examples informing motor-rehabilitation implementation. Non-ASD pediatric, digital-health, and project-management studies are included solely as conceptual or implementation analogues and should not be interpreted as evidence of clinical efficacy for children with ASD. Notes: Introduction: Fidelity, logistical, and training are all important aspects considered by implementation insights, not limited to clinical outcomes. Contact information: Multisite coordination, scalability, and continuing high-quality digital translation lessons. Special lessons learned: Translational lessons in particular contexts or regarding system integrations can be found on several websites. The selection criteria that were used to select studies were different intervention modalities (conventional, play-based, tech-assisted, telehealth, robotics, social-motor programs). This table supplements narrative discussion through the provision of practical implementation information in a succinct format to readers/reviewers.

Study/Year	Sample (n, Age)	Intervention Type	Setting/Multisite Design	Implementation Insights	Key Translational Takeaways
Bhat 2021 [22]	60; 4-12 years	Conventional PT/OT	Single center with home exercise component	Caregiver training influenced adherence to home programs	Early caregiver involvement supports functional gains beyond clinic settings
Bai et al., 2022 [23]	45; 5-10 years	Play-based group motor activities	Three school-based sites	Required coordination across classrooms; variable fidelity observed	Social–motor integration is feasible in school settings with structured staff training
Liu et al., 2022 [24]	32; 6-12 years	Virtual reality-assisted motor training	University laboratory and community clinic	Technology adoption barriers included device availability and staff training	VR may enhance engagement but requires standardized protocols and technical support
Zhang et al., 2022 [25]	50; 4-11 years	Tele-rehabilitation	Four geographically dispersed clinics	Improved reach; challenges related to broadband access and caregiver technology literacy	Hybrid delivery models expand access but require digital equity strategies
Luke et al., 2024 [26]	38; 3-8 years	JASPER-based motor-social intervention	Two clinical sites and one school	Fidelity monitored via video review; weekly inter-site coaching	Centralized supervision supports multisite fidelity and coordination
Bremer and Cairney, 2022 [27]	60; 5-12 years	Robot-assisted upper-limb training	Two rehabilitation centers	Staff cross-training required; high initial setup costs	Robotics may enhance precision practice but scaling requires upfront investment
Zitter et al., 2023 [28]	72; 2-6 years	Early Start Denver Model with motor modules	Three early intervention centers	Centralized data dashboards supported progress monitoring	Shared data systems facilitate multisite fidelity and adaptive planning

Implementation Science in Pediatric Rehabilitation

Multisite implementation involves delivering the same intervention across multiple clinics or service systems while maintaining consistency, quality, and sustainability. In ASD motor rehabilitation, multisite implementation is particularly challenging because programs must align clinical protocols, workforce training, data collection, and family engagement across heterogeneous healthcare, educational, and community settings. Implementation science provides a structured approach for understanding the contextual, organizational, and behavioral factors that influence whether evidence-based interventions are adopted, delivered with fidelity, and sustained over time [29].

Although implementation-science frameworks are increasingly applied in pediatric rehabilitation, their use in ASD motor-rehabilitation research remains limited and often implicit [30]. In this review, these frameworks are discussed specifically in terms of how they inform coordinated multisite delivery, rather than as abstract theoretical models.

Implementation frameworks relevant to multisite ASD motor rehabilitation: Several established implementation frameworks are particularly relevant to multisite ASD motor-rehabilitation programs. The Consolidated Framework for Implementation Research (CFIR) provides a comprehensive taxonomy of determinants across five domains, including intervention characteristics, inner and outer settings, individual characteristics, and implementation processes. For multisite motor-rehabilitation initiatives, CFIR can support pre-implementation readiness assessments, identification of site-specific barriers and facilitators, and selection of tailored implementation strategies over time [31].

The Reach-Effectiveness-Adoption-Implementation-Maintenance (RE-AIM) framework emphasizes population-level impact by examining reach, effectiveness, adoption, implementation fidelity, and maintenance. In pediatric rehabilitation, RE-AIM has been used to assess whether interventions extend beyond controlled trials into routine practice. For ASD motor-rehabilitation networks, RE-AIM indicators can be incorporated into shared monitoring systems to track recruitment, clinical outcomes, site participation, protocol adherence, and long-term sustainability across sites [32,33].

Normalization Process Theory (NPT) focuses on the work required by clinicians, families, and organizations to embed new practices into everyday routines. Applied to ASD motor rehabilitation, NPT can inform strategies to support clinician engagement, integrate motor-rehabilitation protocols into existing service pathways, and sustain collaboration across professional groups and sites [34].

Diffusion of Innovations theory offers a broader perspective on how new practices and technologies spread across systems. This lens is particularly relevant for technology-enabled motor-rehabilitation approaches, where adoption depends on perceived advantage, compatibility with existing workflows, and visibility of benefits [35].

Emerging applications in pediatric and ASD rehabilitation: Although explicit implementation-science applications in ASD motor-rehabilitation remain sparse, related literature in pediatric rehabilitation and autism intervention research provides proof-of-concept for the coordinated model proposed in this review. Scoping reviews have identified increasing use of implementation frameworks to guide education and training strategies for pediatric rehabilitation teams, though reporting quality and consistency remain variable [36]. Recent work in autism intervention research also documents gradual growth in the use of implementation frameworks and hybrid effectiveness-implementation designs, but notes that motor-focused interventions are still underrepresented [37].

The broader rehabilitation and health-services literature provides additional insight. For example, implementation planning tools grounded in CFIR and related frameworks have been developed for clinical trials and large-scale hospital interventions, supporting structured assessment of context, stakeholder engagement, and implementation strategies across multiple centers [38]. Learning collaboratives and quality-improvement collaboratives evaluated using NPT demonstrate how shared governance, iterative feedback cycles, and implementation coaching can support the uptake of complex interventions across diverse sites. Similarly, applications of Diffusion of Innovations to digital health tools illustrate how early adopters, opinion leaders, and robust communication channels can accelerate or impede scaling [39].

While insights from cerebral palsy and other pediatric rehabilitation populations provide valuable guidance for implementation planning, differences in neurodevelopmental trajectories, behavioral regulation, and service pathways in ASD may limit direct generalizability. Recent reviews on integrating implementation science with quality-improvement methods reinforce the need to combine robust theory with pragmatic project-management and data-driven adaptation - an approach closely aligned with the coordinated framework proposed in this article. By explicitly aligning multisite ASD motor-rehabilitation programs with these frameworks, health systems can move from ad-hoc implementation to a deliberate, theory-informed strategy that supports fidelity, scalability, and equity across sites.

Project-Management Methodologies for Multisite Healthcare Implementation

Scaling ASD motor-rehabilitation programs across multiple sites requires not only evidence-based clinical content, but also deliberate coordination of people, processes, timelines, and resources. While implementation science clarifies what factors influence successful uptake and sustainability, project-management methodologies provide practical mechanisms for organizing and operationalizing multisite delivery. Integrating these domains is therefore essential for achieving consistency, scalability, and equity in ASD motor rehabilitation [40].

Project-management approaches supporting multisite ASD motor rehabilitation: In this review, project-management approaches are considered exclusively as supporting infrastructure for multisite implementation, rather than as clinical or efficacy frameworks. Structured methodologies such as PMBOK and PRINCE2 are most relevant for defining shared roles, timelines, accountability structures, and reporting processes across sites [41]. Their primary contribution lies in supporting consistency of non-clinical processes.

Because implementation conditions vary across pediatric rehabilitation settings, adaptive approaches drawn from Agile and Lean methodologies may complement this structure by enabling iterative adjustment of workflows, staffing, and scheduling in response to local constraints [42]. Used selectively, these approaches support local problem-solving while preserving centrally defined clinical standards.

Governance, communication, and stakeholder coordination: Effective multisite implementation of ASD motor-rehabilitation programs depends on clear governance and reliable communication mechanisms. Experience from multisite healthcare and quality-improvement initiatives suggests that a small number of governance components are particularly relevant, including a central coordinating group, site-level implementation teams, shared data and evaluation functions, and structured communication processes [43].

Applied to ASD motor rehabilitation, these mechanisms support consistent recruitment, coordinated training, and fidelity to core intervention components, while enabling timely sharing of implementation challenges and solutions across sites and service sectors [44]. In this review, governance and communication structures are framed as enabling infrastructure rather than prescriptive organizational models.

Cross-sectoral lessons for ASD motor-rehabilitation networks: Evidence from other healthcare and behavioral health domains demonstrates that coordinated project management can support sustained multisite implementation through clear role delineation, shared metrics, and ongoing implementation support [45]. Learning collaboratives illustrate how iterative feedback and joint training can propagate complex interventions across diverse settings.

These cross-sectoral lessons are used here to inform system-level coordination strategies, with the recognition that adaptation is required to account for autism-specific clinical, family, and educational contexts. For ASD motor-rehabilitation programs that incorporate tele-rehabilitation, technology-assisted training, or shared digital dashboards, project-management guidance related to information-system implementation and virtual team coordination is particularly relevant [42].

The coordinated project-management framework for multisite ASD motor rehabilitation: Synthesizing evidence from ASD motor rehabilitation, implementation science, and project-management literature, we propose a Coordinated Project-Management Framework to guide multisite delivery of motor-rehabilitation programs for children with ASD. The framework integrates ASD-specific clinical considerations with implementation constructs and operational coordination into a single applied model.

The framework organizes multisite implementation into five iterative phases: initiation, planning, execution, monitoring and evaluation, and sustainability and scale-up. Each phase embeds implementation-science constructs such as readiness, fidelity, reach, and maintenance within structured project-management processes that define roles, timelines, and accountability across sites.

**Figure 1:**
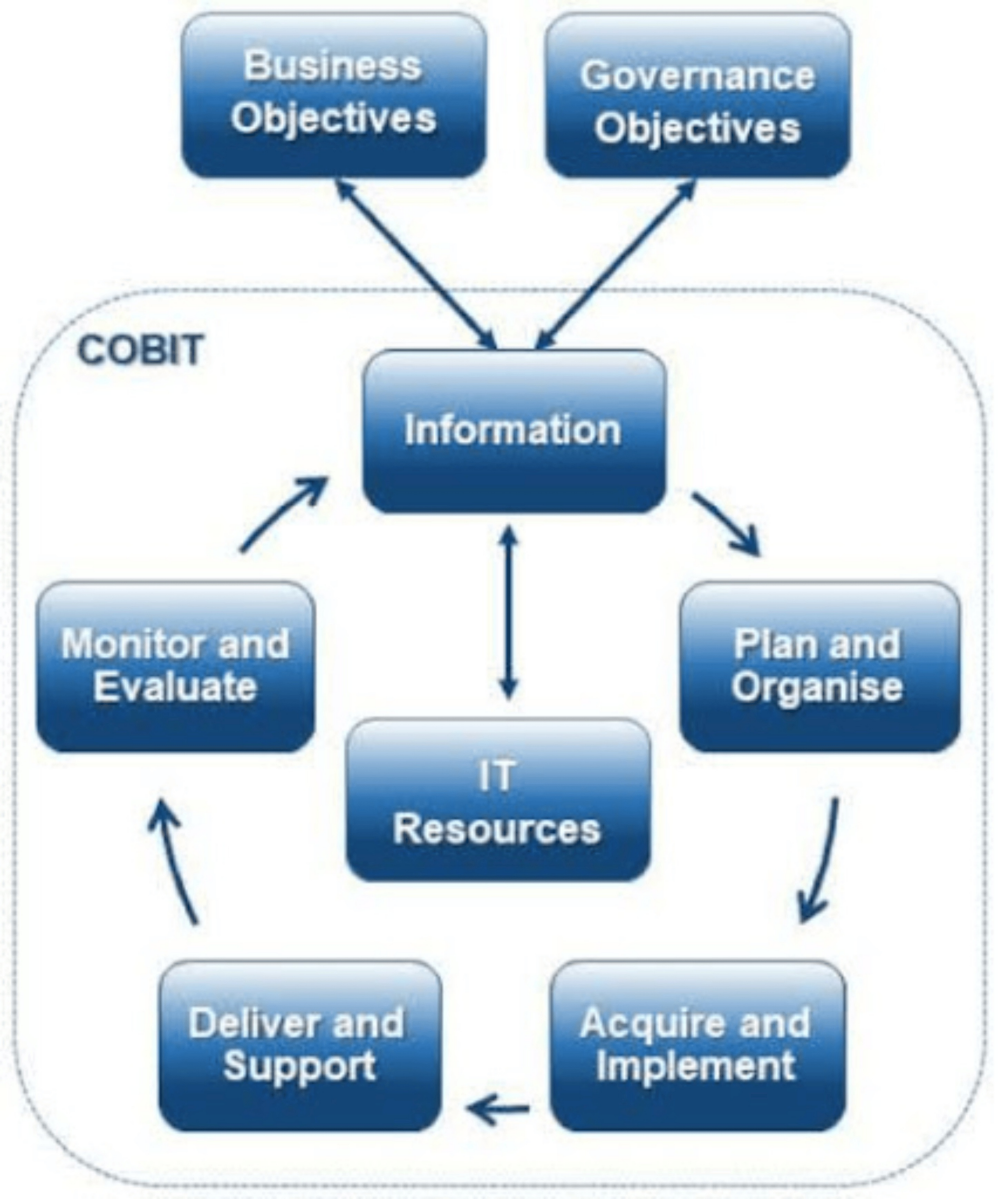
Control Objectives for Information and related Technologies (COBIT) lifecycle model Source: Reference [44]

Figure 1 presents an established lifecycle model from information-technology governance as a conceptual analogue illustrating staged, iterative implementation [46]. The proposed framework builds on similar lifecycle principles but is explicitly tailored to the clinical, organizational, and implementation contexts of ASD motor rehabilitation. It is presented as a conceptual synthesis rather than a direct adaptation of any single existing model.

Project management and digital health literature from non-ASD contexts is included selectively in this review to inform system-level coordination, governance, and scalability strategies relevant to multisite healthcare implementation. These sources are not cited as evidence of clinical effectiveness for children with ASD, but as transferable models for organizing complex, multi-organizational health-service initiatives.

Discussion

The sections above summarize representative evidence on motor impairments in ASD, motor-rehabilitation interventions, and relevant implementation and project-management literature. The Discussion section focuses on integrative synthesis, examining how these domains collectively inform a coordinated approach to multisite motor-rehabilitation implementation.

Taken together, the reviewed literature suggests that the primary barrier to scaling effective ASD motor-rehabilitation interventions is not lack of efficacy, but lack of coordinated implementation infrastructure. Implementation-science frameworks clarify why interventions struggle to move beyond isolated settings, while project-management methodologies provide concrete tools for organizing how multisite programs can be planned, delivered, and sustained. The Coordinated Project-Management Framework integrates these perspectives to address this translational gap.

Thematic Integration and Conceptual Synthesis

Across studies, motor impairments in ASD often co-occur with DCD, ADHD, and sensory integration difficulties are consistently associated with limitations in daily living, participation, and psychosocial functioning. Despite this, motor needs remain under-addressed in many clinical pathways. Evidence from controlled trials and emerging multisite or technology-assisted interventions suggests that structured PT and OT can improve motor proficiency, adaptive behavior, and engagement in meaningful activities [1]. However, heterogeneity in intervention protocols, outcome measures, and follow-up durations limits comparability and hinders replication at scale.

Implementation-science frameworks such as CFIR, RE-AIM, NPT, and Diffusion of Innovations provide complementary perspectives on how to move from isolated efficacy studies to routine, system-wide practice. At the same time, project-management methodologies, including PMBOK, PRINCE2, Agile, and Lean, offer operational tools for coordinating complex multisite initiatives, aligning stakeholders, and monitoring progress against predefined milestones. The proposed Coordinated Project-Management Framework integrates these strands by embedding implementation-science constructs (e.g., context assessment, fidelity, reach, and sustainability) into a structured five-phase lifecycle-initiation, planning, execution, monitoring and evaluation, and sustainability and scale-up [47]. In doing so, it reframes ASD motor-rehabilitation programs not as ad hoc, site-specific efforts, but as coordinated initiatives governed by shared standards, governance structures, and data systems.

Evidence Gaps and Future Priorities

Despite encouraging trends, important gaps remain. First, the evidence base is dominated by small, single-site efficacy studies with variable methodological rigor and limited attention to implementation outcomes. There is a need for pragmatic multisite trials that simultaneously evaluate motor outcomes, feasibility, fidelity, costs, and equity. Such studies should use standardized core outcome sets and common data elements to enable cross-site comparisons and meta-analytic synthesis [48].

Second, digital and data-driven platforms are underutilized. Technology-assisted interventions, such as virtual reality, robotics, and tele-rehabilitation, have demonstrated feasibility and potential benefits for engagement and access, but their integration into coordinated networks is rarely examined. Developing interoperable electronic health record linkages, wearable-sensor pipelines, and centralized dashboards could support real-time fidelity monitoring, adaptive learning loops, and benchmarking across programs [49].

Third, family-centered and culturally responsive models remain insufficiently represented. Many studies do not systematically address caregiver training, language access, or cultural adaptation, despite evidence that early caregiver involvement and community co-design enhance adherence and functional gains. Future models should explicitly incorporate parent-mediated strategies, shared decision-making, and equity metrics, such as differential access, satisfaction, and outcomes across demographic groups, into project charters and performance indicators [50].

Fourth, economic and policy analyses are sparse. Fragmented reimbursement structures, limited autism-specific therapy codes, and state-level variability in Medicaid and telehealth policies continue to constrain scalability. Research linking implementation metrics to value-based care indicators and long-term cost-effectiveness could inform payer incentives and policy reforms supportive of multisite rehabilitation infrastructure [51].

Finally, workforce and training pipelines require systematic development. Current rehabilitation training pathways provide variable exposure to ASD-specific motor and sensory interventions, as well as to implementation science and project-management competencies. Embedding these domains into professional curricula, continuing education, and academic-practice partnerships will be essential for sustaining coordinated networks over time [52].

Feasibility and Scalability of the Proposed Framework

The Coordinated Project-Management Framework is conceptually grounded in models from other multisite health initiatives, including oncology research networks, stroke-rehabilitation consortia, and national behavioral-health collaboratives [52]. These initiatives demonstrate that centrally coordinated yet locally adaptable structures can standardize protocols, harmonize data, and maintain fidelity across diverse settings. Applying similar principles to ASD motor rehabilitation is therefore plausible, provided that context-specific constraints are addressed.

Scalability of the framework depends on three interrelated conditions. First, system integration is needed to align healthcare organizations, educational systems, and community providers through shared governance structures and interoperable data architectures. Aligning motor-rehabilitation plans with Individualized Education Programs and Individualized Family Service Plans may reduce duplication and support continuity across settings. Second, policy and funding alignment must link rehabilitation quality metrics and implementation outcomes to Medicaid waivers, commercial insurance benefits, and federal initiatives such as the Autism CARES Act, ACE programs, and HRSA-supported networks. Third, technological infrastructure must support tele-rehabilitation, sensor-based monitoring, and centralized key-performance-indicator dashboards while ensuring compliance with the Health Insurance Portability and Accountability Act (HIPAA) and the Family Educational Rights and Privacy Act (FERPA) and minimizing digital inequities [53].

When these conditions are met, the framework can function as a learning system in which sites iteratively implement, evaluate, and refine motor-rehabilitation programs while contributing data and lessons to a central coordinating body. This cyclical process aligns with learning health system models and increases the likelihood that effective interventions are sustained and scaled.

Worked Example (Illustrative)

Consider a network of three outpatient pediatric clinics implementing a standardized motor-rehabilitation protocol for children with ASD. During the initiation phase, clinic leaders and therapists collaboratively define core motor targets (e.g., balance, coordination, and functional mobility), eligibility criteria, and shared outcome measures, consistent with early stakeholder-alignment principles emphasized in implementation frameworks [22].

In the planning phase, the network establishes a governance structure with a designated coordinating site, harmonizes therapist training, and selects common assessment tools and data-reporting templates to support consistency and comparability [10,22]. During execution, each clinic delivers the same core intervention components while allowing context-specific adaptations in scheduling and family engagement, reflecting principles of adaptability in multisite implementation [54].

Monitoring and evaluation focus on shared indicators such as reach, fidelity to core components, and changes in standardized motor outcomes, aligned with RE-AIM-informed evaluation approaches [55]. During the sustainability phase, cross-site performance data are reviewed collaboratively to refine workflows, support reimbursement and resource-allocation decisions, and determine expansion to additional sites, consistent with learning-collaborative and maintenance principles [56]. This example illustrates how implementation-science and project-management frameworks can be operationalized in an ASD-specific multisite context informed by motor-impairment research.

Implications for Practice, Systems, and Policy

For clinicians, the proposed framework emphasizes consistent, evidence-informed practice, interdisciplinary collaboration, and explicit attention to participation and quality-of-life outcomes. It encourages routine use of standardized motor assessments, fidelity-monitoring tools, and structured caregiver engagement strategies. For health-system leaders, the framework provides a roadmap for developing multisite implementation capacity through governance structures, project-management support, and integrated data platforms that facilitate quality improvement and research.

At the policy level, coordinated project management offers a mechanism for operationalizing national priorities related to ASD, pediatric rehabilitation, and health equity. Linking multisite motor-rehabilitation initiatives to existing federal and state programs, such as Autism CARES, ACE, Medicaid innovation waivers, and the Individuals with Disabilities Education Act (IDEA) mandates, can incentivize cross-sector collaboration and create more coherent funding and accountability structures. Embedding equity and access metrics into project dashboards and reporting requirements may help ensure that expanded services reach racially, linguistically, and geographically diverse populations [57].

Table 2 summarizes key policy levers relevant to multisite ASD motor rehabilitation and illustrates how the proposed Coordinated Project-Management Framework can be aligned with each.

**Table 2:** A Coordinated Project-Management Framework for multisite motor rehabilitation in autism spectrum disorder (ASD) This table illustrates how key policy levers relevant to ASD motor rehabilitation can be operationalized through the Coordinated Project-Management Framework (CPMF). The framework links policy challenges to concrete governance, implementation, and monitoring mechanisms that support coordinated multisite delivery, sustainability, and equity. RE-AIM: Reach-Effectiveness-Adoption-Implementation-Maintenance; KPI: key performance indicator

Policy Lever	Policy Challenge	How the Framework Plugs In	Illustrative Project-Management Mechanisms
Financing and reimbursement (e.g., Medicaid, commercial insurance)	Fragmented coverage, limited ASD-specific motor-rehabilitation billing pathways, and variability across states	CPMF aligns intervention design with payer requirements and value-based metrics, enabling standardized costing, documentation, and outcome reporting across sites	Standardized project charters; cost and resource tracking; RE-AIM-informed dashboards linking reach, fidelity, and maintenance to reimbursement
Workforce development and training	Uneven therapist preparation in ASD-specific motor interventions; limited exposure to implementation skills	CPMF embeds structured training, coaching, and competency milestones into the planning and execution phases across sites	Centralized training plans; cross-site learning collaboratives; Agile feedback cycles; fidelity audits
Service integration (healthcare-education-community)	Poor coordination between clinics, schools, and early-intervention programs	CPMF provides shared governance and communication structures that align clinical motor goals with educational and community-based plans	Multisector steering committees; shared workflows; coordinated timelines; stakeholder communication plans
Digital health and tele-rehabilitation policy	Variable telehealth authorization, digital inequities, and data-integration barriers	CPMF standardizes platform selection, data governance, and monitoring while allowing local adaptation	Central IT governance; interoperability standards; phased rollout plans; monitoring and evaluation loops
Quality measurement and accountability	Lack of standardized outcomes and implementation metrics across sites	CPMF integrates implementation-science frameworks (e.g., CFIR, RE-AIM) into routine monitoring to support comparability and accountability	Harmonized core outcome sets; fidelity indicators; shared KPI dashboards; continuous quality-improvement cycles
Equity and access mandates	Geographic, socioeconomic, and racial disparities in access to motor-rehabilitation services	CPMF explicitly embeds equity metrics and adaptation strategies into the initiation and sustainability phases	Equity-focused KPIs; stratified reporting; adaptive resource allocation; stakeholder engagement strategies
Sustainability and scale-up incentives	Short-term pilots without long-term continuation or spread	CPMF treats sustainability as a defined project phase, linking policy incentives to maintenance and scale	Sustainability plans; transition-to-operations checklists; diffusion and scale-up roadmaps

Limitations

This review has several limitations. As a narrative, rather than systematic, review, it is subject to publication bias and potential subjectivity in study selection and interpretation, despite efforts to conduct a comprehensive search and apply SANRA quality principles. The integration of literature from rehabilitation science, implementation research, and project management required conceptual synthesis across heterogeneous methodologies and contexts, which may limit direct comparability of findings. In addition, the proposed framework is conceptual and has not yet been prospectively tested as a unified model; empirical validation will require iterative piloting, mixed-methods evaluation, and adaptation to specific health-system environments.

Future Directions

Building on the findings of this review, future work should focus on: (1) designing and evaluating pragmatic multisite motor-rehabilitation trials that incorporate implementation and economic outcomes; (2) developing interoperable digital infrastructures and common data elements for motor outcomes, fidelity, and equity metrics; (3) co-designing family-centered and culturally responsive implementation strategies with diverse communities; (4) integrating implementation science and project-management training into rehabilitation curricula and leadership programs; and (5) piloting the Coordinated Project-Management Framework within existing national or regional networks (e.g., ACE centers, pediatric rehabilitation consortia) to assess feasibility, acceptability, and impact on access and outcomes.

## Conclusions

Motor impairments in children with ASD are common and responsive to intervention, yet motor-rehabilitation services remain fragmented and difficult to scale. This review argues for organizing these services as coordinated, multisite programs rather than isolated clinic-based efforts and introduces a Coordinated Project-Management Framework that integrates implementation-science principles with hybrid project-management approaches to support initiation, planning, delivery, monitoring, and long-term sustainability. Future research should prioritize piloting and evaluating this framework in real-world healthcare networks using hybrid effectiveness-implementation designs to examine clinical motor outcomes, implementation fidelity, and sustainability across diverse settings.
